# Effect of Manual, Electric, and Sonic Toothbrushing on Retention and Surface Integrity of Resin-Based Sealants

**DOI:** 10.1155/ijod/5740355

**Published:** 2025-11-22

**Authors:** Francesco Saverio Ludovichetti, Alessia Rizzato, Valentina Galante, Giovanni Tinazzo, Gabriel Leonardo Magrin, Ernesto Comitale, Edoardo Stellini, Sergio Mazzoleni

**Affiliations:** ^1^Department of Neurosciences, Padova University, Padova, Italy; ^2^Department of Dentistry, Federal University of Santa Catarina, Florianópolis, Brazil

**Keywords:** fissure sealants, shear bond strength, surface roughness, toothbrushing simulation

## Abstract

**Background:**

Fissure sealants are a proven strategy to prevent occlusal caries in children, but their long-term success depends on mechanical stability. While toothbrush efficacy in plaque removal has been extensively studied, its potential impact on sealant retention and surface integrity remains underexplored. This in vitro study assessed the effect of manual, electric, and sonic toothbrushing on the shear bond strength (SBS) and surface roughness of resin-based sealants.

**Methods:**

Sixty flat enamel specimens were obtained from bovine teeth and divided into three groups (*n* = 20) based on brushing modality. Each underwent a standardized 30 min brushing simulation under constant force. SBS was evaluated using a universal testing machine, surface roughness was measured profilometrically, and scanning electron microscopy (SEM) assessed surface changes.

**Results:**

No statistically significant differences in SBS were found among the groups (*p*=0.287). However, significant differences in surface roughness were observed (*p*  < 0.001), with sonic brushing producing the highest roughness values and more pronounced surface degradation on SEM.

**Conclusion:**

Toothbrush type did not affect sealant adhesion under controlled conditions, but sonic brushing increased surface wear. These findings support regular monitoring of sealants in pediatric patients using high-frequency toothbrushes.

## 1. Introduction

Dental caries, both in primary and permanent teeth, is one of the most common diseases globally, affecting all tooth surfaces [1]. Recognized by the World Health Organization as a major public health issue, it remains the most widespread noncommunicable disease, impacting individuals of all ages, genders, and socioeconomic backgrounds. Its high prevalence, especially among children and adolescents, highlights the need for early preventive measures and public health education. Over 520 million children are affected by dental caries in primary teeth [2], a figure that reflects both environmental and behavioral risk factors such as high sugar consumption, poor oral hygiene, and limited access to dental care.

If untreated, dental caries can progressively extend, causing inflammation of the dental pulp and leading to tooth destruction, pain, and eventually extraction [3, 4]. Without timely intervention, this process can result in the development of dental abscesses, systemic infections, and other developmental issues in children. Beyond the immediate health concerns, dental caries significantly impacts the overall quality of life. The pain associated with dental caries can interfere with essential daily activities like eating, sleeping, and learning, particularly in school-age children. It also affects self-esteem [5] and can lead to depression [6], especially in younger populations where appearance and social acceptance are important.

The occlusal surfaces of molars and premolars, with their deep pits and fissures, are particularly susceptible to dental caries [7]. These grooves are prime sites for plaque accumulation, making them a high-risk area for decay. In fact, the complex shape of occlusal surfaces contributes to more than two-thirds of the total caries burden in children [8, 9]. The irregularities in enamel make it difficult to clean these areas effectively during tooth brushing, even in children with good oral hygiene practices. This makes the first permanent molars, and to a lesser extent, the second molars, especially vulnerable to caries during the early years following eruption [10], as the enamel is still maturing and remains more porous and prone to acid attack.

One of the most effective ways to prevent or delay the development of caries in these vulnerable areas is by using fissure sealants [11]. These materials work by sealing the grooves, preventing bacterial buildup and the fermentation of sugars that lead to enamel demineralization. Resin-based sealants, introduced in the 1960s, were the first materials used for this purpose [12]. These sealants form a physical barrier when applied to the occlusal surface following an etching procedure that enhances micromechanical retention [13]. In the 1970s, glass ionomer sealants emerged as an alternative due to their fluoride-releasing properties and their ability to bond chemically without requiring an acid etch [14]. Glass ionomer sealants are particularly useful in environments where moisture control is challenging and contribute to enamel remineralization through continuous fluoride release.

Today, fissure sealants are available in resin-based, glass ionomer-based, and hybrid forms [15]. While resin-based sealants provide excellent retention, they require precise application and complete isolation of the working field. Glass ionomer sealants are easier to apply in moist conditions and offer the added benefit of fluoride release, but they generally have lower mechanical strength. Hybrid sealants aim to combine the best properties of both types, offering better adhesion, wear resistance, and fluoride release.

Numerous longitudinal studies have demonstrated the clinical efficacy of fissure sealants. They consistently report a reduction in occlusal caries of 70%–90% over a period of 2–4 years in sealed teeth compared to unsealed ones [16]. As a result, sealants have become a cornerstone of modern caries prevention, particularly for children and adolescents at high risk for dental decay. However, sealant retention and longevity can decline over time due to normal occlusal forces, abrasion from chewing, and mechanical wear during brushing [17]. Therefore, understanding the factors that influence sealant durability is key to maintaining their effectiveness in preventing caries over the long term.

Toothbrushing remains the primary method for maintaining oral hygiene at home. Its role in disrupting plaque biofilm and promoting gingival health is indispensable. Manual toothbrushes are the most widely used worldwide due to their affordability, availability, and ease of use, and their effectiveness varies depending on technique, duration, and individual dexterity [18].

To address the limitations of manual brushing, powered toothbrushes—both electric and sonic—have been developed to improve plaque removal and user compliance. These devices aim to standardize brushing performance and reduce reliance on brushing technique. Electric toothbrushes, with their small round heads, provide effective mechanical plaque disruption [19]. These brushes often come with features such as pressure sensors, timers, and multiple cleaning modes, making them especially useful for children and individuals with special needs. Sonic toothbrushes, on the other hand, use high-frequency vibrations to create fluid dynamics that help remove plaque even in hard-to-reach areas, improving biofilm disruption. Ultrasonic and multidirectional brushes combine mechanical movement with ultrasonic frequencies to further enhance cleaning efficacy [20]. While promising, there is limited research on how these technologies affect dental materials like fissure sealants. More investigation is needed into the potential impact of sonic toothbrushes on the mechanical stability of resin-based sealants. However, despite the widespread clinical use of powered toothbrushes, there is limited evidence on whether different brushing technologies can influence the mechanical performance and surface characteristics of resin-based sealants. Understanding whether brushing-related mechanical forces affect sealant adhesion or surface integrity is essential, as surface degradation may predispose to plaque retention and early sealant failure.

Therefore, this study aimed to investigate the effect of manual, electric, and sonic toothbrushes on the shear bond strength (SBS) and surface roughness of resin-based sealants under standardized in vitro conditions. The null hypothesis tested in this study was that different toothbrushing technologies (manual, electric, and sonic) would not produce significant differences in the SBS or surface roughness of resin-based sealants.

## 2. Materials and Methods

### 2.1. Specimen Preparation

Sixty bovine permanent incisors [21] were obtained from a certified abattoir and stored in 0.1% thymol solution at 4°C until use. The roots were removed, and the crowns were sectioned using a low-speed diamond saw (Isomet 1000, Buehler, USA) under constant water irrigation to obtain flat enamel blocks (5 mm × 5 mm × 2 mm) from the buccal surfaces. Only blocks with macroscopically intact enamel were selected. The enamel surfaces were polished using 600- and 1200-grit silicon carbide papers to obtain standardized flat surfaces, then ultrasonically cleaned in distilled water for 10 min. A priori sample size calculation was performed using *G*^⁣^*∗*^^Power 3.1 for a one-way analysis of variance (ANOVA), with *α* = 0.05 and power = 80%. An effect size of 0.40 (Cohen's f) was adopted based on previous in vitro studies evaluating the effect of toothbrushing or mechanical wear on resin-based materials, which reported medium-to-large effect magnitudes in roughness changes [20, 22, 23]. This resulted in a required minimum of 18 specimens per group, which was increased to 20 per group to compensate for possible losses.

### 2.2. Sealant Application Protocol

A light-cured resin-based fissure sealant (Clinpro Sealant, 3M ESPE, St. Paul, MN, USA) was applied centrally onto each enamel block using a cylindrical silicone mold (3 mm diameter, 2 mm height) to standardize shape and volume [24]. This material was selected because it is a widely used, well-documented resin-based sealant in pediatric dentistry, with established clinical durability, fluoride-releasing properties, and consistent performance in adhesion studies. Its use in previous laboratory research ensures comparability and methodological continuity with published literature. The enamel was etched with 37% phosphoric acid gel for 30 s, rinsed and air-dried until a frosty white appearance was achieved. The sealant was applied, gently leveled with a probe to avoid bubbles, and polymerized for 20 s with an LED unit (≥1000 mW/cm^2^). After curing, the molds were removed, and the specimens were stored in distilled water at 37°C for 24 h before further testing.

### 2.3. Brushing Simulation Protocol

To simulate clinical toothbrushing and evaluate its mechanical impact on the retention of pit and fissure sealants, all specimens were subjected to a standardized brushing protocol using three different toothbrush systems. Sixty bovine enamel blocks were randomly divided into three experimental groups (*n* = 20 per group), each subjected to a specific brushing modality: Group A (manual toothbrush—Curaprox 5460 ultra soft), Group B (electric toothbrush—Oral-B IO 6, Gentle Care), and Group C (sonic toothbrush—Philips Sonicare Diamond Clean 9000, C2).

Prior to the brushing procedure, all specimens were embedded in cylindrical molds using self-curing acrylic resin (Ortho-Jet, Lang Dental Mfg. Co., Wheeling, IL, USA). This setup allowed for consistent and repeatable contact with the toothbrush bristles during simulation.

Brushing was performed using a customized brushing simulator, adapted to allow consistent control over brushing time, applied force, and motion pattern. The customized brushing simulator was constructed using a mechanical arm with programable linear actuators (Festo ELGR series, Festo AG & Co. KG, Germany), allowing for reproducible horizontal strokes. The apparatus included a force-controlled vertical mounting system equipped with a digital load cell (Mark-10 Series 5, Mark-10 Corp., Copiague, NY, USA) to monitor and maintain a constant applied force of 250 ± 10 g during brushing. For electric and sonic brushes, a 3D-printed adjustable holder was used to fix the devices in the correct orientation, ensuring stable operation throughout the test. All brushing motions were executed automatically by the simulator once configured.

A single trained researcher was responsible for preparing specimens, calibrating the brushing device before each session, and initiating the simulation sequence. The operator also monitored the brushing process in real time to ensure consistent hydration via microrrigation and to replace brush heads every five specimens. No manual brushing was performed directly by the operator. In addition to the three experimental groups, a control group (*n* = 20) consisting of sealant-treated enamel blocks that did not undergo toothbrushing simulation was included. The control specimens were stored in distilled water at 37°C for the same duration as the brushed samples to provide a baseline for comparison of surface roughness and scanning electron microscopy (SEM) morphology.

For the manual group (Group A), brushing was performed using a soft-bristle manual toothbrush (Curaprox 5460 ultra soft, Curaden AG, Switzerland) mounted on a mechanical arm that applied a linear back-and-forth motion (horizontal direction) at a rate of 120 strokes/min. For the electric (Group B) and sonic (Group C) groups, the commercial devices were fixed in position using a 3D-printed holder and operated in active mode, simulating real-use conditions according to the manufacturers' default settings. All groups were brushed for 30 continuous minutes, maintaining a constant vertical force of 250 ± 10 g, verified using a digital force gauge (Mark-10 Series 5, Mark-10 Corp., Copiague, NY, USA). These parameters were selected based on values reported in previous in vitro studies evaluating toothbrush abrasion [20], where similar brushing forces and durations were applied. Although no ISO standard exists specifically for sealant testing, the adopted brushing force of 250 ± 10 g falls within the commonly used range (200–300 g) in simulation protocols. To standardize the environment and eliminate operator variability, all brushing sessions were performed by the same trained researcher using the simulator. The brush heads were replaced every five specimens to ensure consistent bristle integrity and performance throughout the experiment. During brushing, each specimen was kept moist using distilled water delivered via a microirrigation system to simulate the presence of saliva and reduce artificial wear due to dry brushing. A slurry of toothpaste was not used, as the goal of the present study was to isolate the mechanical effect of brushing without confounding factors related to the abrasivity of dentifrice formulations. After the brushing session, all specimens were thoroughly rinsed with distilled water and gently air-dried using oil-free compressed air. The teeth were then stored in distilled water at 37°C for 24 h prior to bond strength testing.

### 2.4. SBS Testing

After the completion of the brushing procedures, all specimens underwent SBS testing to quantitatively evaluate the retention capacity of the fissure sealants following mechanical stress induced by different toothbrush types. This test was conducted using a universal testing machine (Instron 3345, Instron Corp., Norwood, MA, USA), which is commonly employed in dental material testing for its high precision in measuring mechanical properties such as tensile, compressive, and shear strength. Each tooth specimen was embedded in a self-curing acrylic resin (Ortho-Jet, Lang Dental Mfg. Co., Wheeling, IL, USA) using cylindrical molds with an internal diameter of 25 mm and a height of 20 mm. Care was taken to align the long axis of the tooth perpendicular to the base of the mold to ensure uniform load distribution during testing. The samples were then stored in distilled water at 37°C for 24 h prior to mechanical testing to simulate oral environmental conditions. SBS was assessed by applying a vertical force through a custom-made stainless-steel chisel-shaped loading head with a flat contact edge, positioned as close as possible to the interface between the fissure sealant and the enamel surface. The chisel blade was aligned tangentially to the surface, targeting the marginal area of the sealant without damaging the surrounding structure. The load was applied at a crosshead speed of 1.0 mm/min, in accordance with ISO/TS 11405:2015 guidelines for dental adhesive testing, until adhesive failure occurred. The maximum force required to debond the sealant from the enamel surface was recorded in Newtons (N). All tests were carried out in the same laboratory environment by a single calibrated operator to minimize interoperator variability. The testing machine was calibrated according to the manufacturer's specifications prior to the start of the experimental session, and data acquisition was performed using the Bluehill Universal software (Instron Corp., USA). Following the SBS test, all fractured samples were visually examined under 100x and 500x magnification using a stereomicroscope (Leica M80, Leica Microsystems, Wetzlar, Germany). The SBS values obtained were tabulated and subjected to statistical analysis to compare the effect of the different brushing techniques (manual, electric, and sonic) on sealant retention.

### 2.5. Surface Roughness Analysis

After SBS testing, the surface roughness (Ra) of each sealant surface was measured using a contact profilometer (Surftest SJ-210, Mitutoyo Corporation, Kanagawa, Japan). Each specimen was placed on a flat, vibration-free platform, and measurements were performed in a controlled laboratory environment (temperature 22 ± 1°C, relative humidity 50 ± 5%).

For each sample, three consecutive linear scans (length: 4.0 mm; cut-off: 0.8 mm; stylus speed: 0.5 mm/s) were performed at the center of the treated surface, perpendicular to the fissure pattern. The diamond-tipped stylus had a radius of 5 µm and applied a constant vertical force of 0.75 mN. The mean Ra value from the three measurements was recorded for statistical analysis. All measurements were performed by the same trained operator to ensure consistency and repeatability. Calibration of the device was verified using a certified reference specimen before each session.

### 2.6. SEM Analysis

After profilometric measurements, representative specimens from each group (*n* = 3 per group) were prepared for SEM analysis to qualitatively assess surface morphology. Samples were first rinsed with distilled water and air-dried. Dehydration was completed in a desiccator at room temperature for 24 h. The specimens were then mounted on aluminum stubs using double-sided conductive carbon tape and sputter-coated with a 10 nm layer of gold using a Quorum Q150R ES sputter coater (Quorum Technologies Ltd., Laughton, UK) to ensure surface conductivity.

Imaging was performed using a JEOL JSM-IT300 scanning electron microscope (JEOL Ltd., Tokyo, Japan) operating in high vacuum mode. Observations were carried out using secondary electron (SE) detection at an accelerating voltage of 15 kV, a working distance of ~10 mm, and a tilt angle of 0°. Images were acquired at 100x and 500x magnification. All parameters were kept constant across all specimens to allow for valid morphological comparison.

### 2.7. Statistical Analysis

All data were analyzed using R statistical software (version 4.3.1; R Foundation for Statistical Computing, Vienna, Austria). The level of significance was set at *α* = 0.05. All statistical assumptions (normality and homogeneity of variances) were verified before applying parametric tests. Normality of data distribution was assessed using the Shapiro–Wilk test, and homogeneity of variance was evaluated with Levene's test.

For SBS data, a one-way ANOVA was performed to compare mean values among the three groups (manual, electric, and sonic). Since no significant differences were found, post hoc testing was not applied.

For surface roughness values, a one-way ANOVA followed by Tukey's HSD post hoc test was conducted to evaluate pairwise differences between groups.

## 3. Results

The mean SBS values (Newtons), along with the standard deviations, are presented in Table 1.

**Figure 1:**
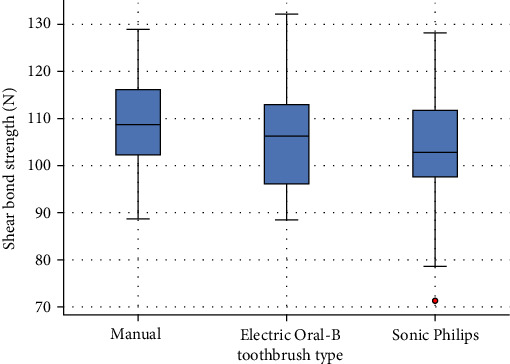
Boxplot showing the distribution of shear bond strength (N) for each toothbrush type. No statistically significant differences were found between groups (ANOVA, *p*=0.287).

The descriptive analysis showed that specimens in Group A (manual) exhibited the highest mean SBS, followed by Group B (electric) and Group C (sonic). Although a decreasing trend in mean values was observed across the groups, the numerical differences did not reach statistical significance. A one-way ANOVA was performed to assess whether the type of toothbrush used influenced the SBS of the sealants (Figure 1). The results indicated no statistically significant difference among the three groups (F[2, 57], *p*=0.287), suggesting that, under the present experimental conditions, the type of brushing modality did not have a measurable impact on sealant retention. Therefore, no post hoc analysis was required. While the data showed a trend toward lower bond strength values in the sonic toothbrush group compared to the manual and electric groups, this difference was not sufficient to support a definitive conclusion. These findings indicate that the mechanical action of brushing—when performed with controlled force, duration, and under standardized conditions—does not appear to significantly compromise the adhesion of fissure sealants. Nevertheless, the slight numerical variation observed across the groups may reflect differences in the mode of mechanical energy transmission, and further research involving larger sample sizes or long-term brushing simulation protocols is warranted to better understand their clinical relevance.

### 3.1. Surface Roughness of Sealants

The descriptive statistics for surface roughness (Ra) are reported below in Table 2:

To assess the differences among the experimental groups (control, manual, electric, and sonic), a statistical analysis was conducted:

The ANOVA revealed statistically significant differences between the groups (*p*  < 0.001). Tukey's test confirmed that all pairwise group comparisons showed significant differences in mean roughness values (*p*  < 0.001, Table 3).

**Figure 2:**
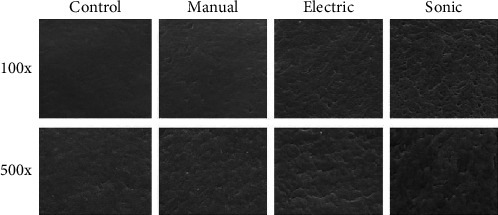
Scanning electron microscope (SEM) images of sealant surfaces on enamel blocks subjected to different brushing methods. The upper row shows surfaces at 100x magnification; the lower row at 500x. From left to right: (1) unbrushed control group, (2) manual toothbrush, (3) electric toothbrush (Oral-B), and (4) sonic toothbrush (Philips Sonicare). A progressive increase in surface roughness and structural degradation is visible from the control to the sonic group, consistent with profilometric measurements of surface wear. Field width at 100x: ~.2 mm; at 500x: ~240 µm.

To further support these quantitative findings, SEM analysis was performed on representative samples from each group. The SEM images provided qualitative insight into the surface topography changes, revealing increasing degrees of surface irregularities and microabrasions in correspondence with the mechanical intensity of the brushing method applied (Figure 2). Based on these results, the null hypothesis was partially rejected: no significant differences in SBS were observed among the three toothbrush types, leading to acceptance of the null hypothesis for this outcome. However, significant differences in surface roughness were detected, resulting in rejection of the null hypothesis for surface roughness.

## 4. Discussion

The present in vitro study was designed to evaluate whether different brushing technologies—manual, electric, and sonic—affect the SBS and surface integrity of a commonly used fissure sealant when applied to enamel. Although no statistically significant differences were found in bond strength among the groups, significant differences were observed in surface roughness values, with sonic toothbrushes producing the most pronounced alterations. Accordingly, the null hypothesis was accepted for SBS and rejected for surface roughness.

Preserving the adhesion and surface quality of sealants over time is critical to ensuring their long-term clinical effectiveness in caries prevention. Although sealants are effective in reducing occlusal caries in children [1, 2], their success depends not only on initial retention but also on resistance to degradation from mechanical forces, including daily toothbrushing. While the literature has extensively covered factors influencing sealant failure—such as operator technique, isolation, and saliva contamination [9]—the potential role of toothbrush type has been less explored. Several previous in vitro studies have evaluated the effect of different toothbrush types on dental materials, although direct comparisons with our study are limited. Wiegand et al. [20] used profilometry and SEM to study abrasion effects on dental hard tissues under varying brushing forces. While their analysis did not include sealants, they reported higher wear in sonic toothbrushes, aligning with our SEM observations of surface degradation. Their statistical approach relied on paired *t*-tests and did not employ ANOVA with post hoc analysis as done in our study.

Our results indicate that, under controlled laboratory conditions, brushing with a sonic toothbrush results in a significantly higher surface roughness compared to both manual and electric brushing. This is consistent with prior studies reporting that sonic brushes, due to their high-frequency bristle movement and generation of hydrodynamic forces, can cause increased surface wear on restorative materials [22]. Although the SBS values showed only a nonsignificant downward trend in the sonic group, the concurrent increase in surface roughness suggests possible early signs of surface fatigue or microdamage.

Surface roughness plays a clinically relevant role in the longevity of sealants. A roughened surface may act as a nidus for plaque accumulation, promoting biofilm maturation and secondary caries formation [25]. It may also compromise the aesthetic quality and stain resistance of the sealant over time. Surfaces with a roughness value (Ra) above 0.2 µm have been shown to retain significantly more plaque and are harder to clean effectively [26]. In our study, all brushing modalities increased the surface roughness compared to the unbrushed control, but this increase was most prominent in the sonic group, as confirmed both by profilometric data and SEM. Although the differences in surface roughness among toothbrush types were statistically significant, their clinical relevance must be interpreted with caution. The brushing duration in this in vitro model represents a short-term challenge compared with cumulative brushing over months or years in real-world conditions. Therefore, while sonic brushing produced higher roughness values, this early effect may not necessarily translate into clinically detectable deterioration in the short term. However, repeated exposure to high-frequency brushing forces could potentially magnify surface changes over time, highlighting the need for long-term evaluation. Consequently, the present findings suggest a possible trend rather than an immediate clinical concern, supporting regular follow-up to monitor sealant surface integrity, especially in children using sonic devices.

SEM analysis revealed a progressive pattern of surface degradation from the control group to the sonic group. While the manual and electric brushes produced surface changes mostly limited to light abrasions and groove marks, the sonic group exhibited widespread microcracks, pitting, and surface porosity. These topographical changes, though subtle, may accelerate material breakdown over time when combined with the thermal, chemical, and enzymatic challenges of the oral environment [21, 27]. Bollen et al. [25] evaluated multiple brushing technologies (oscillatory, linear, sonic, and ultrasonic) and reported variable surface alterations, but their study lacked quantitative bond strength testing. Our study adds to the literature by combining SBS evaluation and surface roughness analysis, using a robust sample size (*n* = 20 per group), consistent brushing protocols, and detailed SEM imaging.

The lack of statistically significant differences in SBS between groups is reassuring from a clinical standpoint, suggesting that the mechanical force generated by different brushing technologies does not immediately compromise sealant adhesion. However, the cumulative effects of mechanical wear, especially from high-frequency brushing, may not be fully captured by a single-cycle debonding test. It is possible that microdamage from daily brushing gradually reduces the interfacial strength or induces marginal breakdown, leading to sealant failure over longer periods.

It is important to note that our brushing protocol was standardized in terms of force (250 g), duration (30 min), and environmental conditions (continuous hydration), allowing for reproducible comparisons between groups. However, in vivo, children may apply greater or inconsistent pressure, and environmental variables such as toothpaste abrasivity, saliva pH, and enzymatic activity can further modify the outcome. Although the exclusion of toothpaste in this study was necessary to isolate the mechanical effect of the brush, it likely underestimates the real-world abrasiveness experienced by sealants during routine hygiene practices [28].

The slight, nonsignificant reduction in bond strength in the sonic group may reflect a more aggressive energy transfer at the bristle-sealant interface. Sonic brushes operate at frequencies of up to 31,000 strokes/min, producing microstreaming effects in the surrounding fluid that increase shear stress [20, 29]. These dynamic forces, although beneficial in plaque removal, could theoretically disturb marginal seal integrity or create subsurface defects over time, especially in materials with lower fracture toughness.

From a clinical perspective, the findings of this study support the continued use of all three types of toothbrushes in children with fissure sealants. However, the increased surface degradation observed with sonic brushes suggests that these devices, while effective in plaque control, may accelerate wear of sealant surfaces. This does not necessarily contraindicate their use but underscores the importance of regular follow-up. Pediatric dentists should monitor sealant integrity at recall appointments, paying attention to discoloration, surface texture, and marginal breakdown—especially in children using high-performance brushes.

Although our findings are derived from a standardized and well-controlled in vitro model, several limitations must be acknowledged. First, the short-term nature of the study does not account for cumulative damage over months or years of use. Second, only one type of sealant material and one model of each toothbrush type were tested; different formulations or brush designs may yield different results. Third, while the brushing simulation was designed to mimic clinical conditions, factors such as salivary enzymes, thermal cycling, dietary acids, and patient-specific brushing habits were not included and could influence outcomes [30, 31].

Future research should focus on long-term brushing simulations, incorporating thermal and chemical cycling, to better understand the durability of sealants in real-world conditions. Additional studies comparing the effects of different bristle stiffness, brush head design, and the interaction with various dentifrice formulations would also help refine guidelines for home care in children with sealants.

### 4.1. Future Directions

Based on the results of this study, future research should aim to validate these in vitro findings under clinical conditions. Longitudinal in vivo studies are needed to assess whether increased surface roughness caused by sonic toothbrushes has a measurable impact on sealant retention, marginal integrity, and caries prevention over time. Additionally, future studies could explore the effects of different sealant formulations, including fluoride-releasing and glass ionomer-based materials, under similar brushing protocols. Investigations incorporating thermal cycling, enzymatic degradation, or the presence of toothpaste abrasives would also provide more realistic simulations of the oral environment. Finally, research comparing the effect of various brushing forces, bristle stiffness, and frequency of brushing could help refine personalized home care recommendations for patients with fissure sealants.

## 5. Conclusions

This study confirms that different toothbrush technologies do not significantly affect the immediate bond strength of sealants under standardized conditions. However, sonic brushing is associated with a greater increase in surface roughness, which could potentially compromise the long-term aesthetic and functional stability of the sealant. Regular clinical monitoring and timely reapplication, when necessary, remain essential components of preventive care in pediatric dentistry.

## Figures and Tables

**Table 1:** Mean shear bond strength values (N) and standard deviations for each group.

Toothbrush type	Mean	Std	Median	Min	Max
Electric	106.59	13.54	107	79	134
Manual	109.45	11.74	109	86	133
Sonic	102.66	15	103	73	133

**Table 2:** Surface roughness (Ra, µm) and statistical comparison among groups.

Group	*n*	Mean Ra (µm) ± SD	Significance letter
Control	20	0.378 ± 0.007	a
Manual	20	0.447 ± 0.010	b
Electric	20	0.547 ± 0.010	c
Sonic	20	0.657 ± 0.010	d

*Note:* Groups sharing different letters differ significantly according to ANOVA and Tukey post hoc test (*p*  < 0.05).

**Table 3:** Post hoc di Tukey HSD test results for surface roughness (mean differences expresses in µm).

Comparison	Mean difference	*p*-Value
Control vs. electric	0.169	<0.001
Control vs. manual	0.069	<0.001
Control vs. sonic	0.279	<0.001
Electric vs. manual	−0.100	<0.001
Electric vs. sonic	0.110	<0.001
Manual vs. sonic	0.210	<0.001

## Data Availability

The data are present in the text.

## References

[1] Kashbour W., Gupta P., Worthington H. V., Boyers D. (2020). Pit and Fissure Sealants versus Fluoride Varnishes for Preventing Dental Decay in the Permanent Teeth of Children and Adolescents. *The Cochrane database of systematic reviews*.

[2] Global Burden of Disease Collaborative Network (2019). Global Burden of Disease Study 2019 (GBD 2019) Results.

[3] Ludovichetti F. S., Stellini E., Rodella C. (2025). Effect of Xylitol and Maltitol Chewing Gums on Plaque Reduction and Salivary pH Modulation: A Retrospective Study in Pediatric Patients. *Dentistry Journal*.

[4] Bukhari O. M. (2020). Dental Caries Experience and Oral Health Related Quality of Life in Working Adults. *The Saudi Dental Journal*.

[5] Kaur P., Singh S., Mathur A. (2017). Impact of Dental Disorders and Its Influence on Self Esteem Levels among Adolescents. *Journal of Clinical and Diagnostic Research*.

[6] Kastenbom L., Falsen A., Larsson P., Sunnegårdh-Grönberg K., Davidson T. (2019). Costs and Health-Related Quality of Life in Relation to Caries. *BMC Oral Health*.

[7] Pitts N. B., Zero D. T., Marsh P. D. (2017). Dental Caries. *Nature Reviews Disease Primers*.

[8] Amend S., Boutsiouki C., Winter J., Kloukos D., Frankenberger R., Krämer N. (2024). Clinical Effectiveness of Pit and Fissure Sealants in Primary and Permanent Teeth of Children and Adolescents: An Umbrella Review. *European Archives of Paediatric Dentistry*.

[9] Wright J. T., Tampi M. P., Graham L. (2016). Sealants for Preventing and Arresting Pit-and-Fissure Occlusal Caries in Primary and Permanent Molars: A Systematic Review of Randomized Controlled Trials-a Report of the American Dental Association and the American Academy of Pediatric Dentistry. *The Journal of the American Dental Association*.

[10] Ramamurthy P., Rath A., Sidhu P. (2022). Sealants for Preventing Dental Caries in Primary Teeth. *Cochrane Database Syst Rev, Feb*.

[11] Wnuk K., Świtalski J., Miazga W., Tatara T., Religioni U., Gujski M. (2023). Evaluation of the Effectiveness of Prophylactic Sealing of Pits and Fissures of Permanent Teeth with Fissure Sealants - Umbrella Review. *BMC Oral Health*.

[12] Ahovuo-Saloranta A., Forss H., Hiiri A., Nordblad A., Mäkelä M. (2016). Pit and Fissure Sealants versus Fluoride Varnishes for Preventing Dental Decay in the Permanent Teeth of Children and Adolescents. *Cochrane Database of Systematic Reviews*.

[13] Eskandarian T., Baghi S., Alipoor A. (2015). Comparison of Clinical Success of Applying a Kind of Fissure Sealant on the Lower Permanent Molar Teeth in Dry and Wet Conditions. *The Journal of Dentistry (Shiraz)*.

[14] Alanzi Abrar N., Muhammad Saleh, Ali Dena, Alomari Qasem (2025). Effectiveness of Dryshield System vs. Cotton Roll Isolation on Sealant’s Retention, Placement Time, and Children’s Acceptance in a Dental School Setting. *Journal of Clinical Pediatric Dentistry*.

[15] Archibald J., Halasa-Rappel Y., Ureles S. D., Miller P., Ng M. W., Sulyanto R. M. (2025). The Association of Sealed Primary Molars With Caries and Restorative Treatments. *Journal of the American Dental Association*.

[16] Crespin M., Iafolla T., Siegal M. D. (2016). Evidence-Based Clinical Practice Guideline for the use of Pit-and-Fissure Sealants: A Report of the American Dental Association and the American Academy of Pediatric Dentistry. *The Journal of the American Dental Association*.

[17] Sarihan Samyeli, Ucuncu Merve Yildirim, Topcuoglu Nursen, Kargul Betul, Eren Figen (2025). *In Vitro* Effects of Different Sugar Alcohol Concentrations on the Cariogenic Biofilm Formed on Fissure Sealants. *Journal of Clinical Pediatric Dentistry*.

[18] Van Der Weijden F., Slot D. E. (2011). Oral Hygiene in the Prevention of Periodontal Diseases: The Evidence. *Periodontology 2000*.

[19] Adam R., Grender J., Timm H., Goyal C. R., Qaqish J. (2025). A 4-Week Randomized Clinical Trial Evaluating Plaque and Gingivitis Effects of a New Oscillating-Rotating Electric Toothbrush. *The Journal of the American Dental Association*.

[20] Wiegand A., Burkhard J. M., Eggmann F., Attin T. (2013). Brushing Force of Manual and Sonic Toothbrushes Affects Dental Hard Tissue Abrasion. *Clinical Oral Investigations*.

[21] Loitongbam M., Mohan R., Chowdhary Z., Mehrotra S. (2020). Comparative Evaluation of Tooth Surface Roughness Caused by Three Different Powered Toothbrushes and a Novel Manual Toothbrush -An SEM and AFM Study. *Indian Journal of Dental Research*.

[22] van der Weijden G. A., Hioe K. P. (2005). A Systematic Review of the Effectiveness of Self-Performed Mechanical Plaque Removal in Adults with Gingivitis Using a Manual Toothbrush. *Journal of Clinical Periodontology*.

[23] Yassen G. H., Platt J. A., Hara A. T. (2011). Bovine Teeth as Substitute for Human Teeth in Dental Research: A Review of Literature. *Journal of Oral Science*.

[24] Mohapatra S., Prabakar J., Indiran M. A., Kumar R. P., Sakthi D. S. (2020). Comparison and Evaluation of the Retention, Cariostatic Effect, and Discoloration of Conventional Clinpro 3M ESPE and Hydrophilic Ultraseal XT Hydro among 12-15-Year-Old Schoolchildren for a Period of 6 Months: A Single-Blind Randomized Clinical Trial. *International Journal of Clinical Pediatric Dentistry*.

[25] Bollen C. M., Lambrechts P., Quirynen M. (1997). Comparison of Surface Roughness of Oral Hard Materials to the Threshold Surface Roughness for Bacterial Plaque Retention: A Review of the Literature. *Dental Materials*.

[26] Lippert V. F., Bresciani E., Mota E. G., Bittencourt H. R., Kramer P. F., Spohr A. M. (2024). In Vitro Comparison of One-Step, Two-Step, and Three-Step Polishing Systems on the Surface Roughness and Gloss of Different Resin Composites. *Journal of Esthetic and Restorative Dentistry*.

[27] Singh T. P., Nirola A., Brar R. (2021). A Profilometric and Scanning Electron Microscopic Analysis of Tooth Surface Abrasion Caused by Rotary/Oscillatory, Linear Motion, Sonic, and Ultrasonic Toothbrushes: An *In Vitro* Study. *Journal of Indian Society of Periodontology*.

[28] Parry J., Harrington E., Rees G. D., McNab R., Smith A. J. (2008). Control of Brushing Variables for the In Vitro Assessment of Toothpaste Abrasivity Using a Novel Laboratory Model. *Journal of Dentistry*.

[29] Digel I., Kern I., Geenen E. M., Akimbekov N. (2020). Dental Plaque Removal by Ultrasonic Toothbrushes. *The Journal of Dentistry*.

[30] Hahnel S., Henrich A., Bürgers R., Handel G., Rosentritt M. (2010). Investigation of Mechanical Properties of Modern Dental Composites after Artificial Aging for 1 Year. *Operative Dentistry*.

[31] Krüger J., Maletz R., Ottl P., Warkentin M. (2018). In Vitro Aging Behavior of Dental Composites considering the Influence of Filler Content, Storage Media and Incubation Time. *PLOS ONE*.

